# Synthesis and Characterization of Composite WO_3_ Fibers/g-C_3_N_4_ Photocatalysts for the Removal of the Insecticide Clothianidin in Aquatic Media

**DOI:** 10.3390/nano14121045

**Published:** 2024-06-18

**Authors:** Christos Lykos, Feidias Bairamis, Christina Efthymiou, Ioannis Konstantinou

**Affiliations:** 1 Department of Chemistry, University of Ioannina, 45110 Ioannina, Greece; c.lykos93@gmail.com (C.L.); bairamisfeidias@gmail.com (F.B.); christinaefthimiou97@hotmail.com (C.E.); 2 Institute of Environment and Sustainable Development, University Research Center of Ioannina (URCI), 45110 Ioannina, Greece

**Keywords:** AOPs, photocatalysis, graphitic carbon nitride, Z-scheme, transformation products, toxicity assessment

## Abstract

Photocatalysis is a prominent alternative wastewater treatment technique that has the potential to completely degrade pesticides as well as other persistent organic pollutants, leading to detoxification of wastewater and thus paving the way for its efficient reuse. In addition to the more conventional photocatalysts (e.g., TiO_2_, ZnO, etc.) that utilize only UV light for activation, the interest of the scientific community has recently focused on the development and application of visible light-activated photocatalysts like g-C_3_N_4_. However, some disadvantages of g-C_3_N_4_, such as the high recombination rate of photogenerated charges, limit its utility. In this light, the present study focuses on the synthesis of WO_3_ fibers/g-C_3_N_4_ Z-scheme heterojunctions to improve the efficiency of g-C_3_N_4_ towards the photocatalytic removal of the widely used insecticide clothianidin. The effect of two different g-C_3_N_4_ precursors (urea and thiourea) and of WO_3_ fiber content on the properties of the synthesized composite materials was also investigated. All aforementioned materials were characterized by a number of techniques (XRD, SEM-EDS, ATR-FTIR, Raman spectroscopy, DRS, etc.). According to the results, mixing 6.5% *^W^*/*_W_* WO_3_ fibers with either urea or thiourea derived g-C_3_N_4_ significantly increased the photocatalytic activity of the resulting composites compared to the precursor materials. In order to further elucidate the effect of the most efficient composite photocatalyst in the degradation of clothianidin, the generated transformation products were tentatively identified through UHPLC tandem high-resolution mass spectroscopy. Finally, the detoxification effect of the most efficient process was also assessed by combining the results of an in-vitro methodology and the predictions of two in-silico tools.

## 1. Introduction

Widespread environmental water pollution due to extensive industrialization and urbanization is a critical issue in the modern era, as available clean water resources are limited [1,2,3]. One of the primary sources of introduction of various pollutants into the aquatic environment is the effluents of wastewater treatment plants (WWTPs) [4,5,6]. This is mainly attributed to the conventional treatment methods applied in these facilities, which are designed to eliminate macropollutants and fail to effectively remove many emerging pollutants (ECs), which consequently end up in environmental water matrices [7,8].

Advanced oxidation processes (AOPs) are non-conventional wastewater treatment methods that can be applied either as pretreatment or ternary treatment stages in WWTPs and are capable of degrading many ECs (e.g., pharmaceuticals, personal care products, pesticides, etc.), ideally converting them to inorganic compounds (e.g., CO_2_, H_2_O, etc.) [9,10]. The various techniques included in the broad category of AOPs generally focus on the in-situ generation of reactive species through physicochemical methods, which then react at high rates and with low selectivity with various ECs to degrade them [11,12].

One of the most widely applied AOP methodologies is heterogeneous photocatalysis, which is based on harnessing solar light to produce reactive species that then degrade ECs via oxidative and reductive pathways [13,14]. Specifically, when a semiconductor particle (photocatalyst) is illuminated by solar light and absorbs a photon whose energy (hv) is greater than its band gap, an electron (e^−^_CB_) is excited and is promoted from the valence band to the conduction band, thereby generating a positive hole (h^+^_VB_) in the valence band [15,16]. The photogenerated h^+^_VB_ and e^−^_CB_ can then either recombine or migrate to the photocatalyst surface via charge transfer interactions and initiate various redox reactions with the adsorbed pollutants, water, and oxygen [17]. Therefore, pollutants can be degraded directly by h^+^_VB_ and e^−^_CB_ or indirectly by reacting with the generated reactive oxygen species (ROS), such as hydroxyl radicals (HO^•^), superoxide anion radicals (O_2_^•−^), singlet oxygens (^1^O_2_), and hydroperoxyl radicals (HO_2_^•^) [9,10,18].

So far, the most common semiconductor utilized in photocatalytic applications, including pollutant degradation, is titanium dioxide (TiO_2_) [19,20]. However, due to its relatively large band gap (E_g_ ≈ 3.2 eV), TiO_2_ can only be photoactivated by UV photons (<387 nm) [21,22]. Considering that UV light constitutes less than 4–5% of the solar spectrum, it becomes apparent that the application of TiO_2_ as a solar photocatalyst is rather limited [23,24]. Therefore, in the last decade, the interest of the scientific community has been focused on the development and application of visible light-responsive photocatalysts such as graphitic carbon nitride (g-C_3_N_4_) and tungsten trioxide (WO_3_) [24,25].

g-C_3_N_4_ is an emerging metal-free 2D π-conjugated polymeric semiconductor composed of tris-s-triazine (heptazine) units [20,26]. The most common methodologies for preparing g-C_3_N_4_ involve the thermal polycondensation of carbon- and nitrogen-rich organic precursors (e.g., urea, thiourea, melamine, and dicyanamide) [27]. As a photocatalyst, it exhibits some attractive features such as easy preparation at low cost, response to visible light (E_g_ ≈ 2.7 eV), favorable electronic band structure, and chemical, photochemical, and thermal stability [28,29]. Despite these characteristics, however, certain limitations (e.g., fast photogenerated charge recombination, low specific surface area, etc.) hinder the photocatalytic performance of g-C_3_N_4_ [23,25].

WO_3_ is a metal oxide n-type semiconductor that, unlike TiO_2_, is visible-light activated due to its narrower band gap (E_g_ ≈ 2.6 eV) that allows it to harness about 12% of the solar spectrum [30,31]. Also, depending on the corner and edge sharing of the WO_6_ octahedra that constitute the WO_3_ crystal, it exhibits several crystal phases, such as tetragonal (α-WO_3_), orthorhombic (β-WO_3_), monoclinic I (γ-WO_3_), triclinic (δ-WO_3_), monoclinic II (ε-WO_3_), hexagonal (h-WO_3_), and cubic (c-WO_3_) [31,32]. Besides the narrow band gap, WO_3_ possesses additional excellent characteristics such as non-toxicity, stability in neutral and acidic aqueous solutions, facile preparation, corrosion resistance, moderate synthesis cost, high valence band energy level (+3.1 eV), and high electron mobility (~6.5 m^2^ (Vs)^−1^), which make it an ideal candidate for photocatalytic applications (e.g., pollutant degradation) [24,33]. However, its low conduction band reduction potential, which does not allow the reduction of oxygen to (O_2_^•−^), as well as the high recombination rate of the photogenerated charges, are two important factors that severely limit the photocatalytic activity of WO_3_ and, by extension, its applicability [34,35].

According to the existing literature, various strategies have been developed to overcome the aforementioned disadvantages of both g-C_3_N_4_ and WO_3_, thereby increasing their photocatalytic activity (e.g., metal/non-metal doping, defect introduction, combination with other semiconductors, etc.) [34,36,37]. Recently, the synthesis of direct Z-scheme heterojunctions has attracted considerable attention in this aspect, as they offer increased light absorption capacity and significant inhibition of the photogenerated charge recombination [28,38]. For direct Z-scheme composite materials to be viable, the band structure of the semiconductive materials of which they are composed must be suitable so that the e^−^_CB_ of the one with the less negative conduction band would recombine with the h^+^_VB_ of the other possessing a valence band with a lower positive potential, just like in the case of WO_3_ and g-C_3_N_4_ [39,40].

In this light, the present study focuses on the synthesis of direct Z-scheme heterojunctions by combining electrospun 1D WO_3_ nanofibers (WOFs) with 2D bulk g-C_3_N_4_ synthesized by using two different precursors (urea and thiourea), as many features of g-C_3_N_4_, such as band gap, surface area, and electron mobility, are precursor dependent [41,42]. The percentage of WOF content (5% and 6.5% *^W^*/*_W_*) in g-C_3_N_4_ was based on the results of our previous work, where a similar approach was utilized to find the optimum amount of WOFs in melamine-derived g-C_3_N_4_ [43]. The structural, morphological, and optical features of all the synthesized materials were characterized by a series of techniques. Also, their ability to generate HO^•^ was investigated via a fluorometric methodology. In order to evaluate the photocatalytic efficiency of the resulting composite materials, the insecticide clothianidin (CLO) was used as a model emerging pollutant. This choice was based on the fact that CLO is a neonicotinoid widely used in various crops worldwide that exhibits high persistence in environmental matrices and is capable of affecting non-target species such as honeybees (*Apis mellifera*) and soil invertebrates [44,45,46,47]. Additionally, the detection of CLO in drainage systems, WWTP effluents, irrigation wells, and wetlands suggests the need to apply non-conventional treatment techniques for its effective removal, like photocatalysis [48,49,50]. In the case of the heterojunction that demonstrated the highest photocatalytic activity, the transformation products (TPs) of CLO formed during the applied process were detected and tentatively identified via ultra-high-performance liquid chromatography tandem high-precision and accuracy mass spectroscopy (UHPLC-LTQ-Orbitrap MS), while the detoxification effect that was achieved was assessed through in-vitro and in-silico approaches.

## 2. Materials and Methods

### 2.1. Chemicals and Materials

Clothianidin reference standard (>99%) was supplied by Dr. Ehrenstorfer GmbH (Ausburg, Germany). Ammonium metatungstate hydrate (99.99%, trace metal basis) (AMH), polyvinylpyrrolidone (M_w_~1,300,000) (PVP), terephthalic acid (98%) (TA), and humic acid (technical) (HA) were obtained from Sigma-Aldrich (St. Louis, MO, USA). Urea (99%) and thiourea (99%) were both provided by Thermo Scientific Chemicals (Waltham, MA, USA). Methanol (HPLC grade), water (HPLC grade), acetonitrile (HPLC grade), methanol (LC-MS grade) (MeOH), and water (LC-MS grade) were all purchased from Fisher Chemical (Loughborough, UK). Sodium hydroxide pellets (analytical grade, ≥98%) (NaOH), hydrochloric acid fuming (37%, for analysis) (HCl), sodium chloride (for analysis) (NaCl), sodium nitrate (for analysis) (NaNO_3_), sodium sulfate anhydrous (for analysis) (Na_2_SO_4_), and formic acid (LC-MS grade, 98–100%) (FA) were supplied by Supelco (Bellefonte, PA, USA). 2-Hydroxyterephthalic acid (≥98%) (2TA-OH) was obtained from Tokyo Chemical Industry (Tokyo, Japan). All chemicals were used as purchased without further treatment. Ultrapure quality water (UPW) was provided on site by an Evoqua water purification system (Pittsburgh, PA, USA). Oasis HLB cartridges (60 mg/3 mL) were obtained from Waters Corporation (Milford, MA, USA).

### 2.2. Preparation of WOFs

WOFs were prepared through a facile method using a typical horizontal electrospinning apparatus. First, 125 mg of AMH were dissolved in 1.25 mL UPW and 200 mg of PVP were dissolved in 2.5 mL of MeOH by vigorous vortexing for 5 min to form a viscous solution. Afterward, the AMH solution was added dropwise to the PVP solution under stirring and the resulting homogeneous viscous solution was introduced to a 10 mL syringe. The filled syringe was then installed on a KDS 100 Legacy Syringe Pump (Holliston, MA, USA), which was appropriately positioned so that the tip of the syringe was 15 cm from a grounded metal drum collector. The feed rate of the syringe was set at 1 mL h^−1^, and the voltage between the syringe tip and the metal drum was maintained at 20 kV throughout the process. Also, relative humidity and temperature were kept constant in the electrospinning area at 28 ± 2% and 30 °C, respectively. The resulting PVP-AMH fibers were then transferred to a capped alumina crucible and calcinated in a muffle furnace at 500 °C for 3 h at a heating rate of 2 °C min^−1^ to finally obtain WOFs after cooling to room temperature.

### 2.3. Preparation of g-C_3_N_4_

Bulk g-C_3_N_4_ derived from either urea (CNU) or thiourea (CNTU) was synthesized via thermal polycondensation. Specifically, 1 g of either urea or thiourea was placed in an alumina crucible semi-covered with a lid. The crucible was then heated in a muffle furnace at 550 °C for 4 h at a heating rate of 10 °C min^−1^, and after cooling to room temperature the resulting CNU or CNTU was collected.

### 2.4. Preparation of WOF/g-C_3_N_4_ Composites

The composite materials were prepared using a simple wet mixing methodology. First, a certain amount (5.0 or 6.5 mg) of WOFs and (95.0 or 93.5 mg) CNU or CNTU was carefully weighted and placed inside a glass beaker. Next, 50 mL of UPW were added to the beaker, and the resulting suspension was bath sonicated for 10 min to achieve better dispersion of the solid particles. The suspension was then magnetically stirred for 2 h followed by heating to 85 °C until dry. Afterward, the resulting solid was transferred to a capped alumina crucible and calcinated in a muffle furnace at 520 °C for 3 h at a heating rate of 5 °C min^−1^. Finally, the composites were cooled to room temperature, collected, and named 5%-WCNU, 6.5%-WCNU, 5%-WCNTU, and 6.5%-WCNTU based on the WOF weight content and g-C_3_N_4_ used.

### 2.5. Characterization Techniques

The X-ray diffraction (XRD) patterns of all synthesized materials were recorded in the 2θ range from 10° to 90° using a Bruker D8 Advance diffractometer (Billerica, MA, USA) with monochromatic Cu-Kα (λ = 1.5406 Å) X-ray radiation. In all cases, the scan rate was set to 0.01° s^−1^.

Attenuated total reflectance Fourier-transform infrared spectroscopy (ATR-FTIR) measurements for both the pristine and composite materials were acquired in the IR region of 4000 cm^−1^ to 400 cm^−1^ utilizing a Shimadzu IR Spirit QATR-S FTIR spectrophotometer (Kyoto, Japan). All spectra were recorded at room temperature with a resolution of 2 cm^−1^.

The Raman spectra of WOFs, CNU, CNTU, 6.5%-WCNU, and 6.5%-WCNTU were obtained using a Horiba Scientific LabRAM Soleil confocal laser Raman microscope (Lyon, France). Specifically, the spectrum of WOFs was recorded in the Raman shift range from 200 cm^−1^ to 1200 cm^−1^ using a 532 nm laser for excitation, while in the case of CNU, CNTU, 6.5%-WCNU, and 6.5%-WCNTU, a 785 nm laser was utilized to overcome the fluorescence interference of g-C_3_N_4_, and the resulting spectra were recorded in the Raman shift region of 400 cm^−1^ to 1800 cm^−1^ [51].

Scanning electron microscopy (SEM) images of the pristine and composite materials were acquired using a Thermo Fisher Pharos Phenom G2 FEG-SEM (Waltham, MA, USA). The instrument was operated under high vacuum (0.1 Pa), using both backscattered and secondary electron detectors in a ratio of 25:75. Also, the electron beam accelerating voltage was set at 15 kV. Additionally, to achieve higher-quality imaging, all samples were first coated with a 5 nm layer of chromium using a Quantum Design Plus sputter coater.

Energy dispersive X-ray spectroscopy (EDS) measurements were also performed for all synthesized materials utilizing a Thermo Fisher Pharos Phenom G2 FEG-SEM (Waltham, MA, USA) under the same conditions applied during SEM imaging.

Adsorption-desorption isotherms of WOFs, CNU, CNTU, 6.5%-WCNU, and 6.5%-WCNTU were recorded at −196 °C (liquid nitrogen) using a Quantachrome Autosorb-1 porosimeter (Bounton Beach, FL, USA). Prior to analysis, a certain amount (~100 mg) of each material was loaded in a 9 mm measuring glass cell with the corresponding rod inserted and degassed under vacuum at 150 °C for 3 h. The specific surface area (S_BET_) of the aforementioned materials was calculated by applying the Brunauer–Emmet–Teller (BET) equation in the relative pressure range of 0.05 to 0.30 [52].

The mean hydrodynamic particle diameter of the pristine and composite materials was determined through dynamic light scattering (DLS) measurements utilizing a Shimadzu SALD-2300 laser diffraction particle size analyzer (Kyoto, Japan).

Diffuse reflectance spectroscopy (DRS) measurements for each synthesized photocatalyst were performed using a Shimadzu UV-2600 spectrophotometer (Kyoto, Japan) equipped with an ISR-2600plus integrating sphere. Reflectance spectra for each material were recorded in the UV-vis region (200–800 nm) and transformed using the Tauc plot, in which the absorption coefficient α was replaced by F(R_∞_) from the Kubelka–Munk function [53]. [F(R_∞_)*hv]^1/γ^ vs. hv was then plotted, and the band gap was determined as the point where the tangent line drawn at the point of inflection of the curve intersected with the x-axis [53,54]. It should be noted that both g-C_3_N_4_ and WO_3_ are considered indirect band gap semiconductors, and therefore, γ was set equal to ½ [55,56]. Also, barium sulfate was used as a reference standard (100% reflectance).

Photoluminescence spectroscopy (PL) measurements were performed for CNU, CNTU, 5%-WCNU, 6.5%-WCNU, 5%-WCNTU, and 6.5%-WCNU utilizing a Horiba FluoroMax-4 spectrofluorometer (Lyon, France). Each material was excited with UV radiation (320 nm) and the resulting emission spectra were recorded in the wavelength range of 400 nm to 600 nm.

### 2.6. Fluorimetric Determination of the Ability to Generate HO^•^

The ability of all synthesized materials to produce HO^•^ photocatalytically was evaluated by observing the transformation of TA (probe) to 2TA-OH for 60 min according to the fluorimetric methodology reported in our previous publication [57].

### 2.7. Photocatalytic Experiments

The photocatalytic activity of all pristine and composite photocatalysts was evaluated through laboratory-scale experiments using an Atlas Suntest XLS+ sunlight simulator (Linsengericht, Germany). First, an aqueous solution of CLO (5 mg L^−1^) was prepared using UPW and transferred to a double-wall Pyrex glass reactor. The reactor was then placed inside the irradiation chamber of the sunlight simulator on top of a magnetic stirrer (300 rpm) and connected to continuous water circulation to maintain its contents at ambient temperature. Afterward, a 5 mL sample of the reactor contents was collected using a plastic syringe and 9.5 mg of solid photocatalyst (100 mg L^−1^) were added. The suspension was allowed to stir in the absence of light for 30 min to reach the adsorption–desorption equilibrium of CLO on the photocatalyst surface. Subsequently, 5 mL of the suspension were sampled and filtered using a syringe disk filter (0.22 μm) to remove the solid photocatalyst, and then the reactor was irradiated (I = 500 W m^−2^, λ > 290 nm) for 240 min. During this 240 min process, 5 mL aliquots of the reactor contents were collected after certain time periods, filtered, and stored in 8 mL glass vials at low temperature (4–8 °C) until further analysis. All photocatalytic experiments were performed in triplicate, and the corresponding percentage relative standard deviation (%RSD) did not exceed 4.5% in all cases, showing the good reproducibility of the applied methodology. Furthermore, it should be noted that the pH in all these experiments was circumneutral (~7).

In the case of the most efficient photocatalyst, further experiments were conducted to evaluate the effect of pH, substrate anions (commonly present in wastewater), and HA acting as dissolved organic matter (DOM). Specifically, two different pH values were selected based on the potential pH range of wastewaters [58]. Depending on the desired value (5 or 9), the pH was adjusted before adding the photocatalyst using either an aqueous solution of HCl (0.1 M) or NaOH (0.1 M). Similarly, the concentration of dissolved anions (10 mg L^−1^) was adjusted per anion case by dissolving a certain amount of the corresponding sodium salt in the reactor solution prior to the addition of the photocatalyst, and the same approach was also used for HA (20 mg L^−1^).

### 2.8. Determination of Residual Concentration of CLO

The residual concentration of CLO in the samples collected during the photocatalytic experiments was determined utilizing a Shimadzu HPLC system (Kyoto, Japan) equipped with an SPD-M40 photodiode array detector. Chromatographic separation of CLO was achieved isocratically using a Supelco Discovery C18 column (15 mm × 4.6 mm, 5 μm particle size) (Bellefonte, PA, USA), while a mixture of water (HPLC) + 0.1% FA and acetonitrile acted as the mobile phase. Also, the column temperature and flow rate were maintained at 40 °C and 1 mL min^−1^, respectively.

### 2.9. In-Vitro Ecotoxicological Assessment with the Microtox Bioassay

The ecotoxicological impact of the photocatalytic processes in which the two most effective composite photocatalysts per g-C_3_N_4_ case were used (i.e., 6.5%-WCNU and 6.5%-WCNTU) was assessed in vitro with the Microtox bioassay using the bacterium *Vibrio fischeri* and an Azur Environmental m500 Analyzer (Carlsbad, CA, USA). The instrument was operated with the MicrotoxOmni v1.18 software and sample evaluation was performed according to the 81.9% Basic Test protocol. Both the bacteria in solid frozen form (Acute Reagent) and the Reconstitution Solution used to activate them were purchased from Modern Water (New Castle, DE, USA). Also, before analysis, a standard phenol solution (100 mg L^−1^) was used as positive control sample and the determined EC_5O_ value was equal to 22 mg L^−1^, thus coming into agreement with the manufacturer’s recommended range (13–26 mg L^−1^).

### 2.10. Sample Precocnetration with Solid-Phase Extraction (SPE)

In order to facilitate the detection and identification of CLO’s TPs, each selected sample collected when 6.5%-WCNU (most efficient) was used as the photocatalyst was preconcentrated via a simple SPE methodology. In brief, five Oasis HLB cartridges (60 mg/3 mL) were inserted onto a Visiprep-DL vacuum extraction manifold and conditioned first with 3 mL of methanol (LC-MS grade) and then with 3 mL of water (LC-MS grade) by applying a flow rate of 1 mL min^−1^. Next, 3 mL of sample were percolated at the same flow rate, and the cartridges were dried under vacuum for 20 min. The retained analytes were then eluted using 2 × 2 mL methanol (LC-MS grade) at 1 mL min^−1^. Finally, the solvent was evaporated under a gentle stream of N_2_ at 40 °C, and the resulting dried samples were reconstituted with 1 mL of methanol.

### 2.11. Detection and Tentative Identification of CLO’s TPs with UHPLC-LTQ-Orbitrap MS

The TPs formed during the photocatalytic removal of CLO using 6.5%-WCNU were detected and tentatively identified utilizing a Thermo Fisher Scientific Accela UHPLC system (Bremen, Germany) coupled to a hybrid LTQ-FT Orbitrap XL 2.5.5 SP1 mass spectrometer equipped with an electrospray ionization source (ESI). Chromatographic separation of CLO and its TPs was carried out using a Thermo Fisher Scientific Hypersil Gold C18 analytical column (100 × 2.1 mm, 1.9 μm particle size) (Bremen, Germany). Elution was achieved using a gradient program, with a mixture of water/0.1% FA (eluent A) and methanol/0.1% FA (eluent B) acting as the mobile phase. The column temperature, flow rate, and injection volume were set at 35 °C, 0.25 mL min^−1^, and 20 μL, respectively. Identification of the aforementioned compounds was performed in positive ionization mode (PI) in the mass range of 90–600 Da, with the mass resolving power set at 60,000 FWHM.

### 2.12. In-Silico Assessment of Ecotoxicologiccal Parameters for CLO and Its TPs

In-silico ecotoxicity estimations for CLO and its tentatively identified TPs at three different trophic levels were performed using the ECOSAR (Ecological Structure–Activity Relationship Model) v2.0 software developed by the United States Environmental Protection Agency (U.S. E.P.A.). Specifically, the software uses quantitative structure–activity relationship (QSAR) models to predict acute (LC_50_ or EC_50_) and chronic (ChV) toxicity values of various compounds to fishes, daphnids, and green algae.

In-silico assessment of mutagenicity and developmental toxicity values for the aforementioned compounds was carried out using the Toxicity Estimation Software Tool (T.E.S.T.) v5.1.2 (also developed by the U.S. E.P.A.), which, like Ecosar v2.0, makes estimations using QSAR models. All evaluations were performed using the consensus method, as this is considered to provide the most accurate predictions according to the T.E.S.T. user’s guide.

## 3. Results and Discussion

### 3.1. Material Characterization

**Figure 1:**
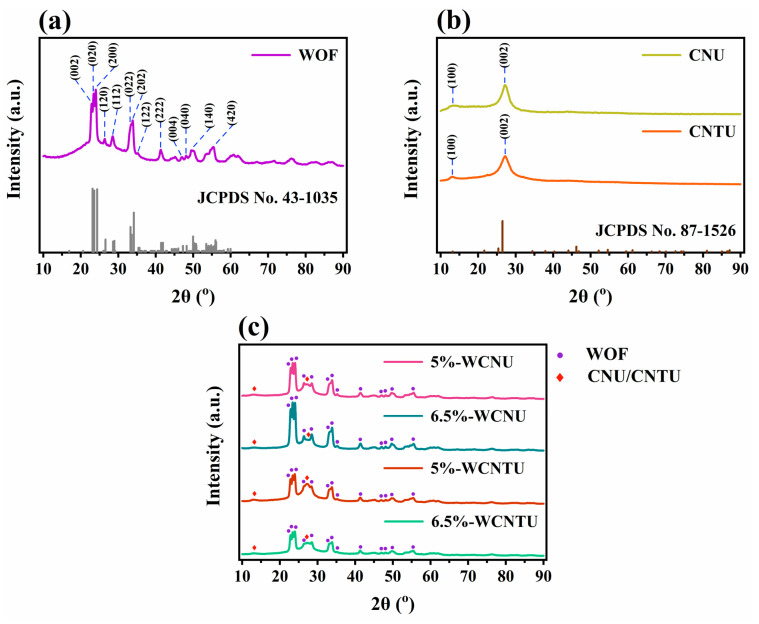
XRD patterns of the synthesized (**a**) WOFs, (**b**) CNU and CNTU, and (**c**) composite materials (5%-WCNU, 6.5%-WCNU, 5%-WCNTU, 6.5%-WCNTU) acquired in the 2θ range of 10–90°.

The crystal structure and phase composition of all synthesized materials were determined through XRD analysis. From Figure 1a, it is evident that the distinct peaks at 2θ = 22.9°, 23.4°, 24.0°, 26.4°, 28.5°, 33.5°, 33.9°, 35.2°, 41.3°, 45.2°, 47.0°, 48.2°, 49.7°, 53.6°, and 55.4°, corresponding to the (002), (020), (200), (120), (112), (022), (202), (122), (222), (132), (004), (040), (140), and (420) lattice planes, respectively, match well with JCPDS Card No. 43-1035, confirming the monoclinic crystalline phase of WOFs with the P21/n space group [59,60,61,62,63]. The average crystallite size for WOFs was calculated by using the Scherrer equation for the aforementioned 2θ values and found to be 15.80 nm [64,65,66,67]. These findings are consistent with the crystallographic features of electrospun WO_3_ fibrous materials reported in the literature, as they all exhibited a monoclinic crystalline phase with average crystallite sizes ranging from 13.00 to 26.50 nm [59,66,67,68].

According to the diffraction patterns of CNU and CNTU (Figure 1b), both materials displayed two distinct broad peaks, one of low intensity at 2θ = 13.1° and one of much higher intensity at 2θ = 27.2° and 27.1°. These peaks are well matched with JCPDS Card No. 87-1526 and are characteristic of g-C_3_N_4_ [69,70]. Specifically, the one at 2θ = 13.1° is assigned to the (100) lattice plane, which is related to the in-plane repetitive structural packing motif of the tris-s-triazine units, while the one at 2θ ≈ 27° corresponds to the (002) lattice plane and is associated with the graphite-like stacked layers of g-C_3_N_4_ consisting of conjugated aromatic units [71,72,73,74,75]. By applying Bragg’s law for the peaks located at 2θ = 27.2° and 27.1°, the interlayer spacing for CNU and CNTU was calculated and found to be equal to 0.328 nm and 0.329 nm, respectively [73,76,77]. This slightly denser structure of CNU compared to CNTU could be potentially attributed to the presence of oxygen-containing molecules produced only during the thermal polycondensation of urea that act as leaving motifs, promoting the condensation process [42,78].

The XRD patterns of all composite materials as presented in Figure 1c demonstrated a diffraction pattern similar to that of WOFs, while two additional peaks, one of very low intensity and one of moderate intensity, could be distinguished at 2θ ≈ 13° and 27°, respectively. The existence of these two peaks confirms the presence of either CNU or CNTU and by extension the successful synthesis of the heterojunctions. Despite the relatively low content of WOFs in the composite materials, it appears that the extensive dispersion of fibers on the surface of CNU and CNTU significantly reduced the intensity of the characteristic peaks associated with g-C_3_N_4_ in the resulting diffraction patterns [79].

**Figure 2:**
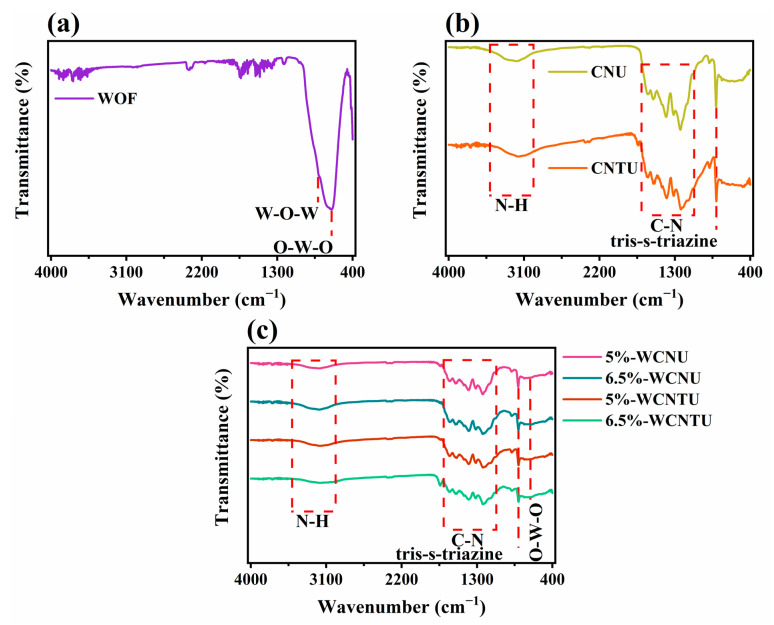
ATR-FTIR spectra of the synthesized (**a**) WOFs, (**b**) CNU and CNTU, and (**c**) composite materials (5%-WCNU, 6.5%-WCNU, 5%-WCNTU, 6.5%-WCNTU) recorded in the wavenumber region of 4000–400 cm^−1^.

The chemical composition of the synthesized pristine and composite materials was studied through ATR-FTIR spectroscopy. As can be observed from the ATR-FTIR spectrum of WOFs (Figure 2a), in the broad area ranging from 480 to 1025 cm^−1^, two peaks could be identified. Specifically, the most intense band centered at 668 cm^−1^ is ascribed to the W-O-W stretching modes, while the less intense one at 802 cm^−1^ is assigned to the O-W-O stretching modes [80,81]. Both of these peaks are characteristic of WO_3_, and their presence is also stated in other studies, which report the synthesis of WO_3_ fibrous materials under similar electrospinning/annealing conditions [80,81,82,83].

The ATR-FTIR spectra of CNU and CNTU (Figure 2b) exhibited a very high similarity, confirming that these materials had practically the same chemical structure despite the use of different precursors. In both spectra, the sharp band located at ~807 cm^−1^ is attributed to the out-of-plane bending vibrations (also known as breathing mode) of the tris-s-triazine (or s-triazine) units [41,75]. Also, the less intense band centered at ~888 cm^−1^ is assigned to the N-H deformation mode of the cross-linked tris-s-triazine, while the bands located in the broad region from 1000 cm^−1^ to 1650 cm^−1^ are associated with the characteristic skeletal stretching vibrations of the C-N heterocycles [73,84]. The distinct bands centered around 1316 cm^−1^ and 1231 cm^−1^ can be attributed to the out-of-plane bending vibrations of tri-s-triazine units connected via trigonal N-(C)_3_ or C-NH-C bridging units, which are indicative of complete and incomplete polycondensation of the melem monomers, respectively [41,85]. Moreover, the existence of a broad band ranging from 3000 cm^−1^ to 3500 cm^−1^, which is characteristic of N-H stretching vibrations, is indicative of the presence of residual or terminating amino groups in the structure of g-C_3_N_4_ [75,84].

In the case of the composite materials, all the characteristic bands attributed to the chemical structure of g-C_3_N_4_ were clearly distinct, as shown in Figure 2c. Furthermore, the band located at ~660 cm^−1^, indicative of the presence of WO_3_, was not visible due to the rather low weight content of WOFs in the composites. 

**Figure 3:**
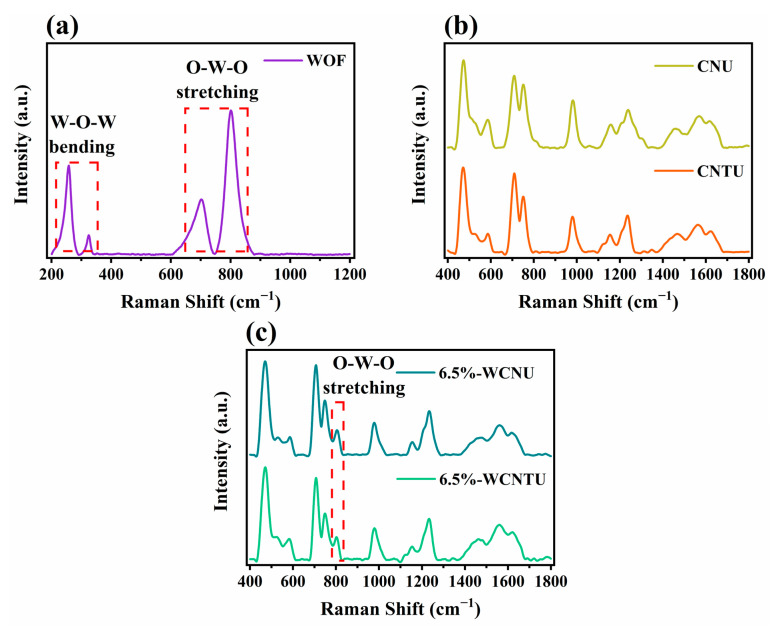
Raman spectra of the synthesized (**a**) WOFs (532 nm), (**b**) CNU and CNTU, and (**c**) composite materials (5%-WCNU, 6.5%-WCNU, 5%-WCNTU, 6.5%-WCNTU) (785 nm).

The phase composition and characteristic bonds of all synthesized materials were further investigated by Raman spectroscopy. In the Raman spectrum of the WOFs (Figure 3a), four characteristic peaks could be clearly distinguished. The two peaks centered at 258 cm^−1^ and 325 cm^−1^ are related to the W-O-W bending vibrations of the bridging oxygen, while the peaks located at 702 cm^−1^ and 801 cm^−1^ are ascribed to the asymmetric (longer bonds) and symmetric (shorter bonds) O-W-O (W^6+^-O) stretching modes, respectively [65,86,87,88]. These peaks are indicative of the monoclinic crystalline phase of WOFs and are in agreement with the aforementioned XRD results [65,86,87].

The Raman spectra of CNU and CNTU presented in Figure 3b demonstrated peaks located at 473 cm^−1^, 586 cm^−1^, 709 cm^−1^, 752 cm^−1^, 983 cm^−1^, 1153 cm^−1^, and 1428 cm^−1^, all of which are associated with the characteristic vibration modes of the tris-s-triazine heterocycles that make up g-C_3_N_4_ [51,89]. Specifically, the most intense peak at ~473 cm^−1^ is assigned to the in-plane (twisting) vibrations of the tris-s-triazine heterocycles, while the peaks centered at ~709 cm^−1^ and ~983 cm^−1^ are ascribed to the two different breathing modes of the s-triazine rings [90]. Additionally, the peaks located at ~752 cm^−1^ and ~1248 cm^−1^ are related to layer-to-layer (deformation) vibrations of C-N heterocycles and the =C (sp^2^) bending vibrations, respectively [90,91,92]. All these structural features of CNU and CNTU are in agreement with the results of ATR-FTIR spectroscopy.

Examining the Raman spectra of the composite materials with the highest WOF content (i.e., 6.5%-WCNU and 6.5%-WCNTU), it becomes apparent that they both exhibited high similarity to the spectra of CNU and CNTU. However, their common peak located at ~801 cm^−1^ is characteristic of the presence of WOFs in both, indicating that these materials were successfully synthesized.

**Figure 4:**
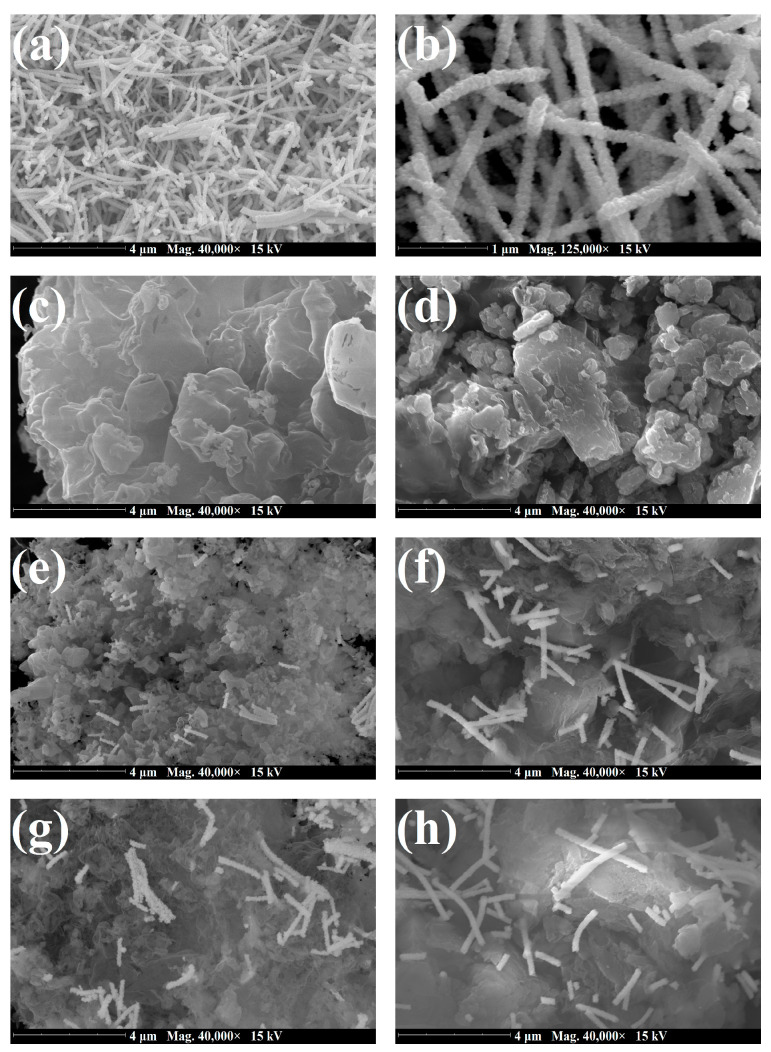
SEM images of (**a**,**b**) WOFs, (**c**) CNU, (**d**) CNTU, (**e**) 5%-WCNU, (**f**) 5%-WCNTU, (**g**) 6.5%-WCNU, and (**h**) 6.5%-WCNTU, acquired using an accelerating voltage of 15 kV under high vacuum (0.1 Pa).

The morphological features of the pristine and composite materials were examined by SEM imaging. As presented in Figure 4a,b, the as-prepared WOFs had a rough surface and were composed of small bead-like particles. Furthermore, it can be observed that their diameters varied between 180 and 230 nm. Similar characteristics have also been reported for WO_3_ fibrous materials that were synthesized via electrospinning approaches [68,81]. The micrographs of CNU and CNTU (Figure 4c,d) show that both materials consisted of stacked aggregated sheets (flakes), which is typical for g-C_3_N_4_ [74]. In the case of composite materials (WCNU), their respective images (Figure 4e–h) show that all were composed of WOFs that were dispersed unevenly over CNU and CNTU flakes. In addition, agglomerates of a few WOFs could also be observed.

**Figure 5:**
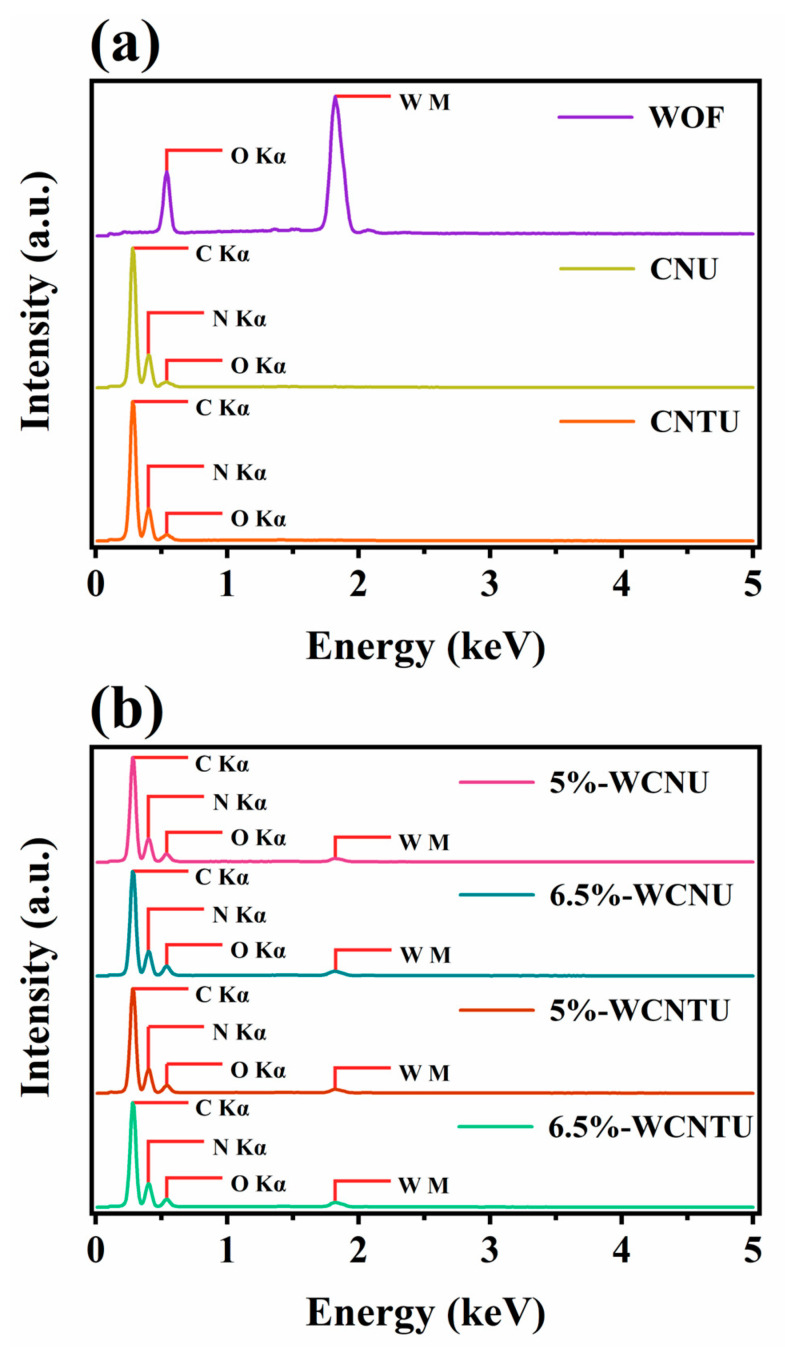
EDS spectra of the synthesized (**a**) pristine (WOFs, CNU, and CNTU) and (**b**) composite materials (5%-WCNU, 6.5%-WCNU, 5%-WCNTU, and 6.5%-WCNTU), recorded using an accelerating voltage of 15 kV under high vacuum (0.1 Pa).

EDS analysis of WOFs (Figure 5a) confirmed the presence of both oxygen (O Ka~0.53 KeV) and tungsten (W M~1.78 KeV) atoms, with an atomic ratio (W:O) of 1:3. In addition, the EDS spectra for CNU and CNTU confirmed that both materials were composed only of carbon (C Ka~0.28 KeV) and nitrogen (N Ka~0.39 KeV), while oxygen was also present at very low concentrations, possibly due to oxygen-containing terminations and/or CO_2_ adsorbed from the atmosphere. According to the EDS spectra for the composite materials, the amount of tungsten in 6.5%-WCNU and 6.5%-WCNTU was about 1.3 times higher than that of 5%-WCNU and 5%-WCNTU, respectively, proving that after the synthetic process, all the composites seemed to contain the desired quantity of WOFs.

**Figure 6:**
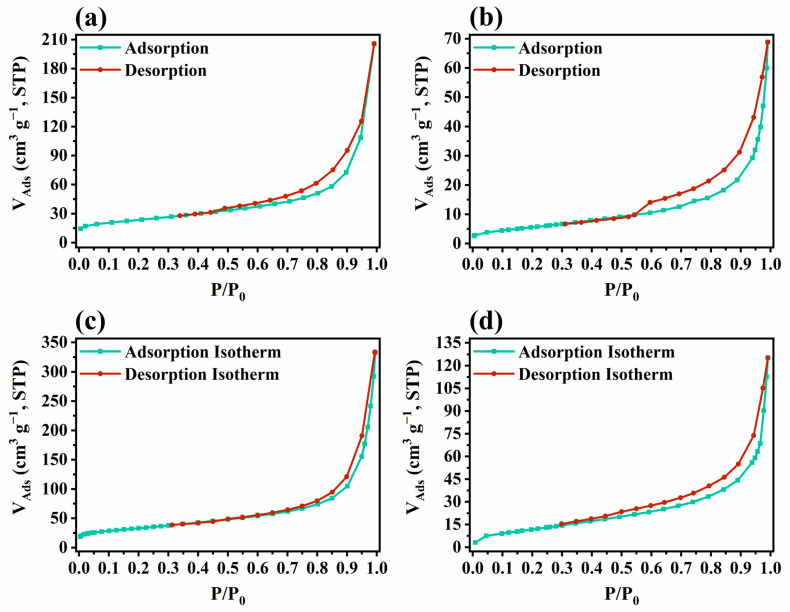
Adsorption–desorption isotherms of (**a**) CNU, (**b**) CNTU, (**c**) 6.5%-WCNU, and (**d**) 6.5%-WCNTU recorded at −196 °C.

The adsorption–desorption isotherms of CNU, CNTU, 6.5-WCNU, and 6.5-WCNTU are presented in Figure 6. According to the IUPAC technical report on physisorption, all these isotherms are classified as Type IVa with H3 hysteresis loops and are typical for mesoporous materials [52,93]. From the application of the BET equation, the S_BET_ for CNU, CNTU, 6.5-WCNU, and 6.5-WCNTU was calculated to be 82.29 m^2^ g^−1^, 46.65 m^2^ g^−1^, 106.29 m^2^ g^−1^, and 47.46 m^2^ g^−1^, respectively. The observed difference in S_BET_ between CNU and CNTU has also been reported in other studies and can potentially be attributed to the formation of CO_2_ (only during the thermal polycondensation of urea), which is chemisorbed onto basic docking sites, inhibiting the formation of large grains [41,78,93]. Interestingly, the introduction of WOFs in both CNU and CNTU resulted in a slight increase in S_BET_, as in other cases of WO_3_/g-C_3_N_4_ heterostructures, suggesting an increase in the number of active sites where adsorption can take place [94,95].

The mean hydrodynamic diameters for CNU, CNTU, 5%-WCNU, 6.5%-WCNU, 5%-WCNTU, and 6.5%-WCNTU were estimated to be 314 nm, 343 nm, 294 nm, 323 nm, 297 nm, and 319 nm, respectively. These data indicate that when thiourea was used as a precursor, the resulting CNTU particles tended to have a slightly larger size when dispersed in water compared to those derived from urea. Furthermore, the fact that the composites exhibited smaller hydrodynamic diameters compared to the pristine materials could be attributed to the partial thermal exfoliation of g-C_3_N_4_ due to the calcination step involved in the synthesis process [73,75].

**Figure 7:**
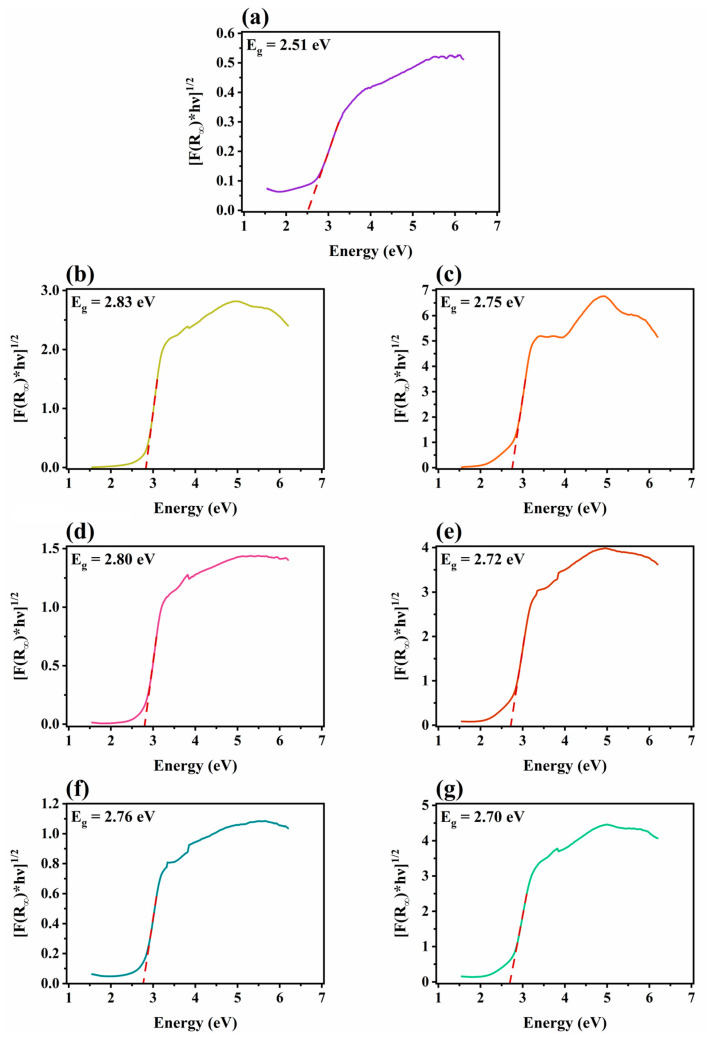
[F(R_∞_)*hv]^1/2^ vs. hv plots for (**a**) WOFs, (**b**) CNU, (**c**) CNTU, (**d**) 5%-WCNU, (**e**) 5%-WCNTU, (**f**) 6.5%-WCNU, and (**g**) 6.5%-WCNTU, and corresponding determined band gaps.

The optical band gaps of all synthesized materials were calculated from the corresponding [F(R_∞_)*hv]^1/2^ vs. hv plots presented in Figure 7. As expected, all photocatalysts were capable of absorbing light in the blue region of the UV-vis spectrum. In addition, it appears that CNU exhibited a slightly larger band gap compared to CNTU, which is consistent with various reports from the literature and could be attributed to quantum confinement effects arising from the different degree of condensation of urea compared to thiourea [42,93,96]. All composite photocatalysts demonstrated narrower band gaps than their corresponding g-C_3_N_4_ precursors, and it appears that a higher WOF content led to a further increase in the light absorption ability of the synthesized photocatalysts. This red shift of the absorption edge was also reported for other WO_3_/g-C_3_N_4_ heterojunctions, and it results from the interactions between the two individual semiconductors [93,97].

**Figure 8:**
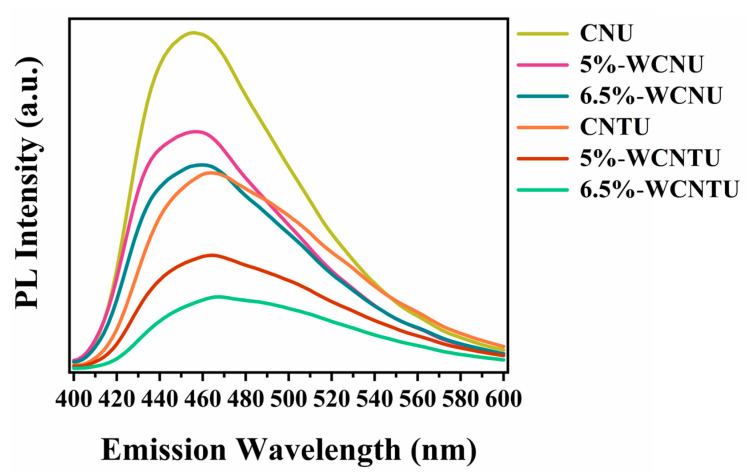
PL spectra of CNU, CNTU, 5%-WCNU, 5%-WCNTU, 6.5%-WCNU, and 6.5%-WCNTU (excitation wavelength: 320 nm).

PL studies are important for determining the rate at which the photogenerated charges recombine in a semiconductor [57]. From the PL spectra presented in Figure 8, it can be clearly observed that CNTU exhibited a lower PL intensity compared to CNU, which is consistent with the findings of similar works and probably results from the existence of a larger number of structural defects (such as uncondensed amino groups) in the structure of CNTU, which act as electron captors, facilitating charge separation [42,98]. In the case of composite photocatalysts, a similar profile was observed, with the increase in WOF weight content leading to a further decrease in PL intensity in the corresponding materials. The lower PL intensity of the composite photocatalysts is attributed to the successful formation of Z-scheme heterojunctions that promote efficient charge separation [97,99].

### 3.2. Study of CLO Degradation Kinetics and HO^•^ Generation Ability of the Synthesized Photocatalysts

**Figure 9:**
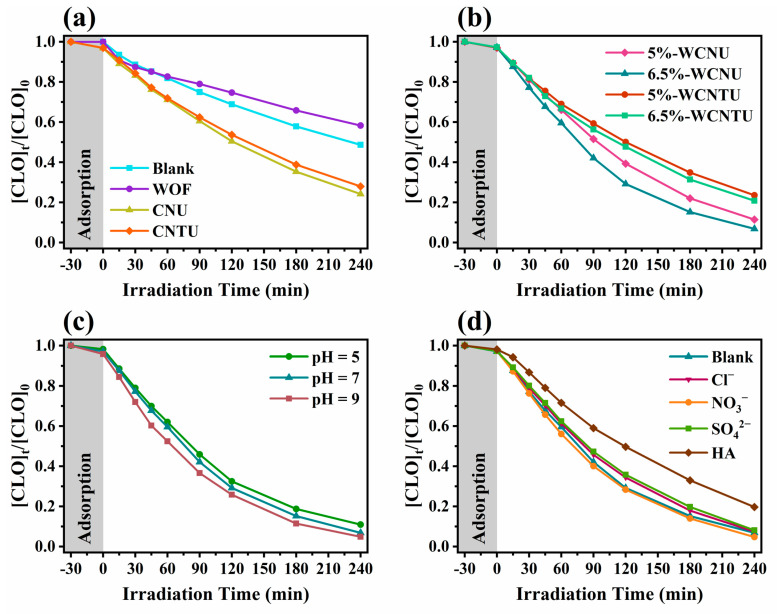
(**a**) Photocatalytic degradation kinetics of CLO (5 mg L^−1^) using the synthesized pristine (CNU, CNTU) and (**b**) composite photocatalysts (5%-WCNU, 6.5%-WCNU, 5%-WCNTU, 6.5%-WCNTU) (100 mg L^−1^) under simulated sunlight (500 W m^−2^). (**c**) Effect of different pH values on the degradation kinetics of CLO (5 mg L^−1^) using 6.5%-WCNU (100 mg L^−1^) under simulated sunlight (500 W m^−2^). (**d**) Effect of different anions and humic acid (acting as dissolved organic matter) on the degradation kinetics of CLO (5 mg L^−1^) using 6.5%-WCNU (100 mg L^−1^) under simulated sunlight (500 W m^−2^).

The photocatalytic activity of all the prepared pristine and composite materials was evaluated by utilizing them for the photocatalytic degradation of the insecticide CLO. In addition, to assess the effect of solar light on the degradation of CLO, experiments were conducted in the absence of a photocatalyst using the same methodology. The resulting degradation kinetics presented in Figure 9a,b were fitted in a pseudo-first-order kinetic model, and the corresponding kinetic data are included in Table 1. 6.5%-WCNU was the composite that exhibited the highest photocatalytic activity despite its slightly wider band gap and higher charge recombination rate compared to CNTU, 5%-WCNTU, and 6.5%-WCNTU. Various studies involving the use of urea- and thiourea-derived g-C_3_N_4_ as a photocatalyst have reported similar degradation kinetic results, as in all cases CNU showed higher photocatalytic activity than CNTU [41,100,101,102]. The most probable explanation for these observations is the superior ability of CNU (compared to CNTU) to facilitate the migration of the photogenerated charges at the active sites located on the photocatalyst surface, as it exhibits a higher photocurrent density according to photocurrent measurements [42,102,103,104]. Therefore, in the present work, CNU and, by extension, CNU-based composites all exhibited better performance in the degradation of CLO than their CNTU counterparts. Interestingly, WOFs were found to be less efficient even than direct photolysis, possibly due to the high rate of charge recombination. Moreover, the WOF particles blocked the simulated solar light, inhibiting the photolytic process that occurs simultaneously with photocatalysis.

**Figure 10:**
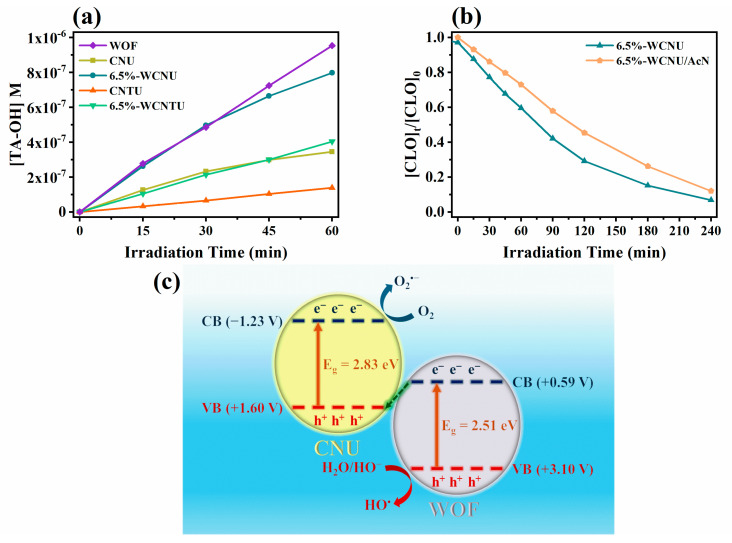
(**a**) Photocatalytic evolution kinetics of 2TA-OH using the synthesized pristine (CNU, CNTU) and composite photocatalysts (6.5%-WCNU, 6.5%-WCNTU) (100 mg L^−1^) under simulated sunlight (500 W m^−2^). (**b**) Photocatalytic degradation kinetics of CLO (5 mg L^−1^) using 6.5%-WCNU (100 mg L^−1^) in AcN under simulated sunlight (500 W m^−2^). (**c**) Schematic representation of the Z-scheme photocatalytic mechanism in the 6.5%-WCNU.

The evolution kinetics of 2TA-OH (Figure 10a) show that 6.5%-WCNU exhibited the highest yield in HO^•^ compared to 6.5%-WCNTU, CNU, and CNTU. Despite the relatively low WOF content of 6.5%-WCNU, the corresponding 2TA-OH kinetic profile closely matched that of pristine WOFs, further indicating that the resulting direct Z-scheme heterojunction had the ability to separate the photogenerated charges more effectively, facilitating the generation of HO^•^ either directly through the oxidation of water by the h^+^_VB_ of WOFs [33] or indirectly through a reductive pathway utilizing the e^−^_CB_ of CNU to produce O_2_^•−^, which is then ultimately converted into HO^•^ [105]. The fact that WOFs demonstrated the highest HO^•^ yield and achieved the slowest kinetics suggests that CLO probably reacted to a lesser extent with HO^•^. To further elucidate this matter, a photocatalytic experiment was conducted using 6.5%-WCNU and replacing UPW with AcN, as the absence of water was expected to severely decrease the generation of HO^•^ since under these conditions it can only be produced via a reductive pathway, as previously mentioned. The results summarized in Figure 10b and Table 2 clearly show that this approach resulted in a slight inhibition in the removal of CLO, confirming the above statement. All these findings are in agreement with the results of scavenging studies conducted in another work, which indicated that the photocatalytic removal of CLO is primarily dominated by O_2_^•−^ despite the use of TiO_2_-based photocatalysts that are known for their superior HO^•^ production [106].

Considering that 6.5%-WCNU demonstrated the highest photocatalytic efficiency towards the removal of CLO, the effect of pH on the overall process was investigated. As shown in Figure 9c and Table 2, by lowering the pH to 5, the removal of CLO was slightly reduced, while the opposite effect was observed for pH = 9. The most probable factor that contributed to the slightly higher removal of CLO at pH = 9 was the increased concentration of HO^−^, as it can be oxidized by h^+^_VB_ of WOFs to yield HO^•^. The above results show that 6.5%-WCNU is a photocatalyst that maintained its photocatalytic activity in a broad pH range, thus suggesting that it can be utilized for the removal of ECs (such as CLO) in wastewater whose pH is usually in the range of 5–9, as previously mentioned.

The presence of either Cl^−^ or SO_4_^2−^, according to Figure 9d, appeared to slightly inhibit the degradation kinetics of CLO, as both of these anions reacted with HO^•^ and h^+^_VB_ to generate their respective radicals, which have a lower oxidation potential than the aforementioned reactive species [107,108]. Therefore, these anions can act as scavengers for HO^•^ and h^+^_VB_, thus decreasing the photocatalytic efficiency of the applied process. Additionally, both Cl^−^ and SO_4_^2−^ can be adsorbed by 6.5%-WCNU, reducing the available active sites for the adsorption of CLO. Interestingly, when NO_3_^−^ was added to the solution, the overall removal of CLO demonstrated an increase. Although, like the other two anions, it was expected to act as scavenger of both HO^•^ and h^+^_VB_, it appears that its ability to also generate HO^•^ upon exposure to solar light in the presence of hydrogen cations (H^+^) was the main reason behind the observed results, which indicate that the latter phenomenon may have been more dominant [107,109,110]. Finally, HA was the substrate that provided a significant inhibitory effect, as CLO removal was ~1.7 times slower. According to scavenging studies on the photocatalytic removal of HA conducted in another study, it appears that its removal is mainly mediated by O_2_^•−^ and to a lesser extent by HO^•^ and h^+^_VB_ [111]. Therefore, these findings suggest that CLO and HA potentially compete with each other in order to react with O_2_^•−^.

It should be noted that during the photocatalytic removal of CLO with 6.5%-WCNU Cl^−^, NO_3_^−^ and SO_4_^2−^ are expected to form. However, since the complete mineralization of CLO could ideally yield 0.7 mg L^−1^ of Cl^−^, 6.2 mg L^−1^ of NO_3_^−^, and 1.9 mg L^−1^ of SO_4_^2−^, which are lower than the concentrations in the aforementioned experiments, their effect on the degradation kinetics of CLO would be negligible.

Based on all the above conclusions, the photocatalytic direct Z-scheme mechanism presented in Figure 10c can be proposed. According to the existing literature, CNU exhibits a valence band potential of about 1.6 V vs. NHE, while WO_3_, as previously mentioned, has a valence band potential of approximately 3.1 V vs. NHE [33,112,113,114]. Therefore, the respective conduction band potentials are calculated by substracting the determined E_g_ values and are equal to −1.23 V for CNU and +0.58 V for WOFs. After the irradiation of these two semiconductors with solar light (hv > E_g_), the photogenerated e^−^_CB_ of WOFs will recombine with the h^+^_VB_ of CNU due to the lower energy difference between their respective bands, and thus, superior charge separation is achieved [39,43]. As a result, the e^−^_CB_ of CNU can react with the adsorbed oxygen to produce O_2_^•−^ due to their favorable reduction potential, while the h^+^_VB_ of WOFs can directly oxidize water to generate HO^•^.

### 3.3. In-Vitro Assessment of Ecotoxicity Changes

Although AOPs are generally considered efficient techniques for the removal of various toxic compounds, in some cases they can potentially lead to the formation of TPs, which are more toxic than their parent compound [14,115]. Therefore, evaluating the ecotoxicological impact of such processes is of utmost importance to determine whether they lead to less or more toxic effluents.

**Figure 11:**
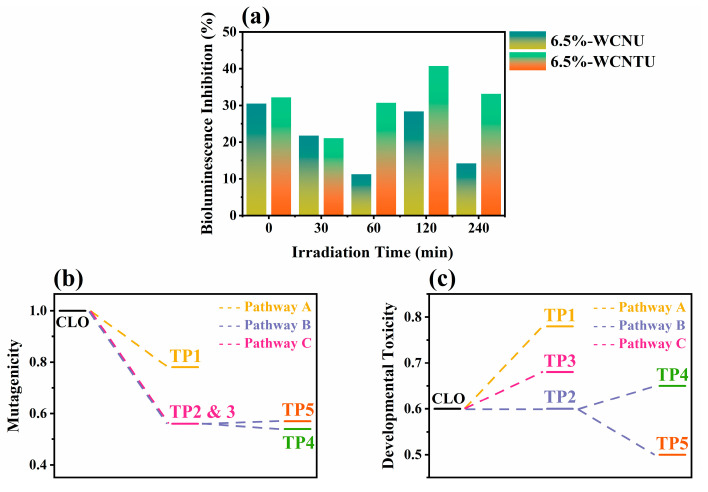
(**a**) Microtox bioassay results when 6.5%-WCNU and 6.5%-WCNTU were utilized and (**b**,**c**) in-silico predicted mutagenicity and developmental toxicity values for CLO and its TPs formed when 6.5%-WCNU was used.

Since 6.5%-WCNU and 6.5%-WCNTU demonstrated the highest photocatalytic activity compared to CNU and CNTU, respectively, the ecotoxicty changes in the processes in which these two materials were used were monitored with the Microtox bioassay. From Figure 11a, it is evident that the application of 6.5%-WCNU significantly reduced the toxicity of the CLO-containing solution within the first 60 min. However, after 120 min of irradiation, an increase was observed, possibly due to the formation of either toxic TPs or synergistic effects between existing TPs. Interestingly, at the end of the applied photocatalytic process with 6.5%-WCNU, the total ecotoxicity was reduced to less than half of the initial ecotoxicity. According to Figure 11a, when 6.5%-WCNTU was utilized, a slight decrease in the ecotoxity was achieved within the first 30 min. Within the next 90 min, the % bioluminescence inhibition increased to 40.71%, indicating a considerable increase in the overall ecotoxicity, and then started to decrease again until the end of the photocatalytic process. These results suggest that longer irradiation time periods are required in order to achieve a higher detoxification effect when 6.5%-WCNTU is used, which is reasonable since it exhibited slower degradation kinetics compared to 6.5%-WCNU. Variations in ecotoxicity (bioluminescence inhibition) were also observed in a study investigating the photolytic removal of CLO (3 mg L^−1^) under simulated sunlight [116]. Furthermore, in another work in which TiO_2_ was utilized for the photocatalytic removal of CLO from distilled water, it is stated that after 120 min of irradiation, an increase in the bioluminescence inhibition of *Vibrio fischeri* was noticed, as in the case of 6.5%-WCNTU [117].

### 3.4. Detection and Identification of CLO’s TPs

In order to further elucidate the effectiveness of 6.5%-WCNU in the degradation of CLO, the TPs formed during the photocatalytic process were detected and tentatively identified via UHPLC-LTQ-Orbitrap MS. A total of five TPs were identified, and their corresponding high-resolution mass spectroscopy data are summarized in Table 3. It should be noted that the identification of all the aforementioned TPs was based on their chromatographic (retention) behavior, accurate mass, pseudo-molecular ion, and MS^2^ fragmentation ions, as well as the results of similar studies where possible [57]. Furthermore, the confidence level (CL) of each identification was defined according to the work of Schymanski et al. [118].

**Figure 12:**
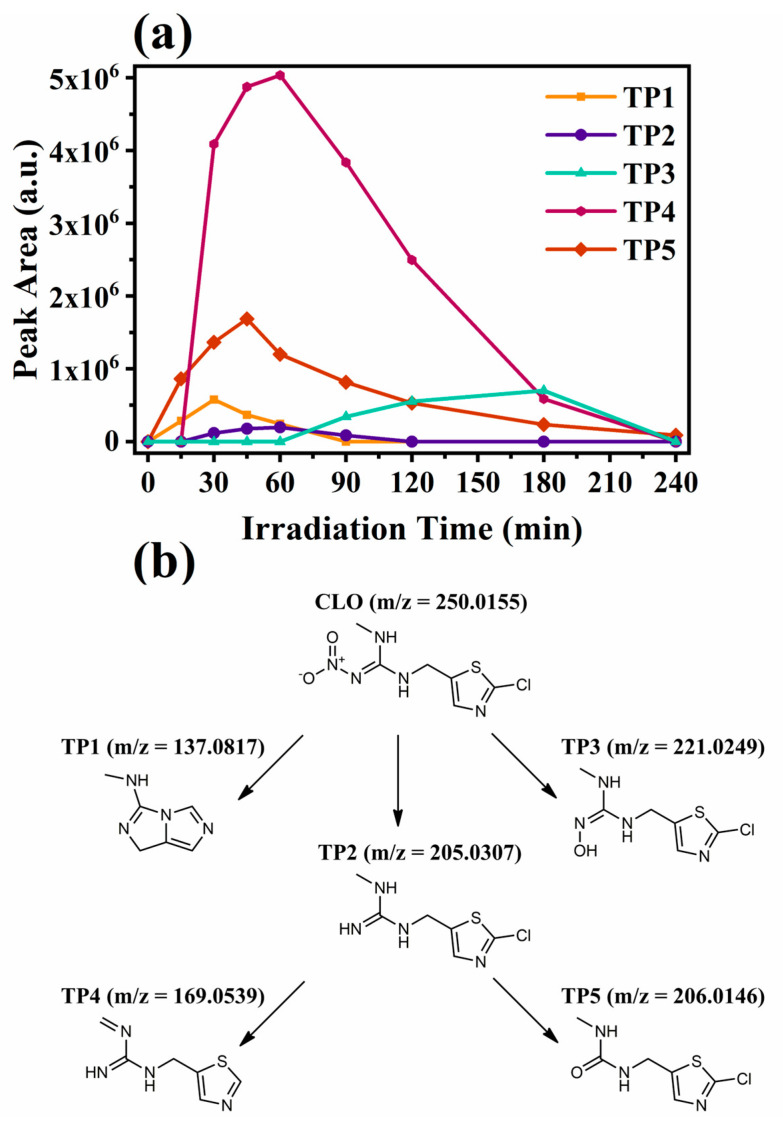
(**a**) Evolutionary profiles of CLO’s tentatively identified TPs based on high-resolution MS data and (**b**) the proposed transformation pathways of CLO.

TP1 (*m*/*z* = 137.0817 Da) showed a mass difference of 112.9338 Da from the parent compound’s pseudo-molecular ion, suggesting the loss of both chlorine and nitro groups as well as a subsequent opening of the thiazole ring and a loss of the sulfur atom towards the formation of a final bicyclic compound. Therefore, it was tentatively identified as N-methyl-1H-imidazo[1,5-c]imidazol-3-amine (CL: 3). The mass difference of the pseudo-molecular ion of TP2 (*m*/*z* = 205.0307 Da) and that of CLO was equal to 44.9848 Da, which is indicative of the loss of a nitro group. Therefore, it was probably identified as 1-((2-chlorothiazol-5-yl)methyl)-3-methylguanidine (CL: 2). Photolytic studies on the degradation of CLO have also identified TP2 as a photodegradation product [116,119]. TP3 (*m*/*z* 221.0249 Da) appears to be the substitution of the product CLO’s nitro group by a hydroxyl group, according to the pseudomolecular mass difference (28.9906 Da) and MS^2^ fragmentation ions. Based on these findings, it was tentatively identified as (E)-1-((2-chlorothiazol-5-yl)methyl)-2-hydroxy-3-methylguanidine (CL: 3). TP4 (*m*/*z* 169.0539 Da) exhibited a mass difference of 80.9616 Da from the ion of CLO, which is indicative of the loss of both the chlorine group and the nitro group accompanying the formation of a double bond. As a result, it was tentatively identified as 1-methylene-3-(thiazol-5-ylmethyl)guanidine (CL: 3). The pseudo-molecular ion of TP5 (*m*/*z* = 206.0146 Da) differed from the pseudo-molecular ion of the parent compound by 44.0009 Da, suggesting the loss of the =N-NO_2_ group followed by the addition of oxygen in its place. It was probably identified as the 1-((2-chlorothiazol-5-yl)methyl)-3-methylurea (CL: 2), and according to other works, it can be formed during photolytic or photocatalytic activity (with TiO_2_) the removal of CLO from aqueous matrices [116,119,120]. All five TPs were also identified in our previous publication on the photolytic removal of CLO using hydrochar [44]. Furthermore, based on their structure and evolutionary profiles (Figure 12a), three possible transformation pathways are proposed and are presented in Figure 12b.

### 3.5. In-Silico Evluation of the Ecotoxicity of CLO and Its TPs

The ecotoxicity of CLO and its tentatively identified TPs to fish, daphnids, and green algae was predicted using Ecosar v2.0. Based on the resulting acute and chronic toxicity values, each compound was classified as either very toxic (LC_50_, EC_50_, ChV ≤ 1 mg L^−1^), toxic (1 mg L^−1^ < LC_50_, EC_50_, ChV ≤ 10 mg L^−1^), harmful (10 mg L^−1^ < LC_50_, EC_50_, ChV ≤ 100 mg L^−1^), or not harmful (LC_50_, EC_50_, ChV ≥ 100 mg L^−1^) to the corresponding trophic level according to the Globally Harmonized System of Classification and Labeling of Chemicals (GHS) [121]. Also, to make these data (to some extent) comparable with the Microtox bioassay results, LC_50_ values for daphnids were mainly assessed, as the marine bacterium *Vibrio fischeri* is considered biologically more similar to this class of zooplanktonic crustaceans compared to fish and green algae [122].

The in-silico predicted toxicity values for CLO and its tentatively identified TPs are summarized in Table 4. Of all the TPs, only TP1 and TP5 were estimated to be less toxic to daphnids than CLO. Interestingly TP2, TP3, and TP4 when classified as nicotinoids (NIs) presented much lower LC_50_ values (at the same trophic level) than CLO when it was categorized as a neonicotinoid (NN). However, when all these compounds were classified as aliphatic amines (AAs), only TP4 was found to be significantly more toxic than the parent compound, while TP2 and TP3 showed slightly lower and considerably higher LC_50_ values for daphnids, respectively. Since CLO belongs to the neonicotinoid class, the results corresponding to this as well as the broader nicotinoid class were taken into account for the assessment. From the evolutionary profiles (Figure 12a) of TP2, TP3, and TP4, it becomes clear that by the end of the photocatalytic process they were all degraded. Therefore, it could be hypothesized that the low bioluminescence inhibition observed after 240 min of illumination (Figure 11a) could actually be attributed to the residual concentration of CLO and TP4, with the most probable scenario being the potential synergistic effect between them or with other non-identified TPs present at very low concentrations.

In the case of mutagenicity, the in-silico predicted values for CLO as well as its tentatively identified TPs showed that all these compounds were classified as “mutagenicity positive” (mutagenicity value > 0.5). However, as presented in Figure 11b, all the proposed pathways led to the formation of TPs that were much less mutagenic than the parent compound. Therefore, the photocatalytic treatment of CLO with 6.5% WCNU can potentially have positive results in this regard, as it addresses a serious ecotoxicological risk.

Finally, according to developmental toxicity prediction (Figure 11c), both CLO and its proposed TPs were characterized as “developmental toxicants” since their respective estimated values were higher than 0.5. The suggested pathways in the majority of the generated compounds are more likely to negatively affect the growth of organisms than CLO, and it was hence concluded that the applied photocatalytic technique did not prove effective in this respect.

## 4. Conclusions

In summary, composite direct Z-scheme WO_3_ fibers/g-C_3_N_4_ photocatalysts were successfully fabricated using facile green synthesis methods. The effect of the precursor in the synthesis of g-C_3_N_4_ and by extension the resulting composites was also investigated, as both urea and thiourea were used. The structural, morphological, and optical properties of all synthesized materials were characterized by a number of spectroscopic and microscopic techniques. The 6.5%WCNU exhibited the highest efficiency in both the generation of HO^•^ and the degradation of CLO compared to the other composites. These findings were attributed to the effective separation of the photogenerated charges due to the successful formation of the Z-scheme heterojunction. The ecotoxicity evaluations revealed that the application of 6.5%-WCNU for the photocatalytic removal of CLO is a viable alternative, as the process significantly decreased the toxicity and led to the formation of TPs, which in their majority were predicted to be less toxic than the parent compound. In conclusion, it appears that the easy fabrication of direct Z-scheme heterojunctions through methods that can be employed on an industrial scale is a viable approach for enhancing the characteristic of traditional semiconductors, which as standalone materials face many limitations, prohibiting their application in larger-scale processes.

## Figures and Tables

**Table 1:** Percent adsorption at equilibrium, degradation kinetic constants (k_app_), correlation coefficients (R^2^), calculated half-lives (t_1/2_), and percentage removals of CLO (5 mg L^−1^) using the synthesized photocatalysts (100 mg L^−1^) under simulated solar light (500 W m^−2^).

Photocatalyst	Adsorption (%)	k_app_ (min^−1^)	R^2^	t_1/2_ (min)	Removal (%)
Blank	-	0.0031	0.9976	224	51.3
WOFs	0.1	0.0024	0.9756	289	41.7
CNU	3.2	0.0056	0.9984	124	75.8
5%-WCNU	3.0	0.0083	0.9900	84	88.6
6.5%-WCNU	2.8	0.0104	0.9912	67	93.2
CNTU	3.0	0.0051	0.9994	136	72.0
5%-WCNTU	2.9	0.0058	0.9992	120	76.5
6.5%-WCNTU	2.7	0.0063	0.9988	110	79.2

**Table 2:** Percent adsorption at equilibrium, degradation kinetic constants (k_app_), correlation coefficients (R^2^), calculated half-lives (t_1/2_), and percentage removals of CLO (5 mg L^−1^) using 6.5%-WCNU (100 mg L^−1^) in the presence of Cl^−^ (10 mg L^−1^), NO_3_^−^ (10 mg L^−1^), SO_4_^2−^ (10 mg L^−1^), HA (20 mg L^−1^), AcN (solvent), or different pH values under simulated solar light (500 W m^−2^).

Conditions	Adsorption (%)	k_app_ (min^−1^)	R^2^	t_1/2_ (min)	Removal (%)
Blank	2.8	0.0104	0.9912	67	93.2
pH = 5	1.7	0.0090	0.9956	77	88.9
pH = 9	3.1	0.0118	0.9955	59	95.0
Cl^−^	2.5	0.0098	0.9899	84	92.6
NO_3_^−^	2.5	0.0114	0.9908	61	95.3
SO_4_^2−^	2.7	0.0094	0.9896	74	91.9
HA	1.9	0.0062	0.9935	112	80.3
AcN (solvent)	2.7	0.0078	0.9792	89	88.0

**Table 3:** Chromatographic and high-resolution mass data of CLO and its TPs formed during the photocatalytic process using 6.5%-WCNU.

Compound	t_R_ (min)	[M + H]^+^/[M + Na]^+^	Molecular Formula	Δ (ppm)	RDB	MS^2^ [M + H]^+^	Molecular Formula	Δ (ppm)	RDB
CLO	7.30	250.0155	C_6_H_9_O_2_N_5_ClS	−2.358	4.5	220.0170	C_6_H_9_ON_4_ClS	−4.731	4.0
						206.0144	C_6_H_9_ON_3_ClS	−2.752	3.5
						204.0226	C_6_H_9_N_4_ClS	−2.432	4.0
						169.0537	C_6_H_9_N_4_S	−2.920	4.5
						168.0460	C_6_H_8_N_4_S	−2.550	5.0
						131.9665	C_4_H_3_NClS	−2.986	3.5
TP1	0.83	137.0817	C_6_H_9_N_4_	−3.522	4.5	81.0440	C_4_H_5_N_2_	−8.943	3.5
TP2	2.07	205.0307	C_6_H_10_N_4_ClS	−1.226	3.5	188.0038	C_6_H_7_N_3_ClS	−3.044	4.5
						169.0537	C_6_H_9_N_4_S	−3.215	4.5
						163.0086	C_5_H_8_N_2_ClS	−3.210	2.5
						148.9930	C_4_H_6_N_2_ClS	−3.176	2.5
						131.9664	C_4_H_3_NClS	−3.971	3.5
						113.0162	C_4_H_5_N_2_S	−5.269	3.5
TP3	0.66	221.0249	C_6_H_10_ON_4_ClS	−4.189	3.5	185.0486	C_6_H_9_ON_4_S	−3.016	4.5
						168.0458	C_6_H_8_N_4_S	−3.681	5.0
						164.9879	C_4_H_6_ON_2_ClS	−2.956	2.5
						131.9663	C_4_H_3_NClS	−4.729	3.5
						129.0112	C_4_H_5_ON_2_S	−3.954	3.5
						113.0161	C_4_H_5_N_2_S	−6.154	3.5
TP4	0.71	169.0539	C_6_H_9_N_4_S	−2.210	4.5	113.0161	C_4_H_5_N_2_S	−6.154	3.5
TP5	6.23	206.0146	C_6_H_9_ON_3_ClS	−1.490	3.5	174.9723	C_5_H_4_ON_2_ClS	−2.502	4.5
						148.9929	C_4_H_6_N_2_ClS	−3.847	2.5
						131.9664	C_4_H_3_NClS	−3.971	3.5
						119.9663	C_3_H_3_NClS	−5.202	2.5
						113.0162	C_4_H_5_N_2_S	−5.269	3.5

Retention time (t_R_), pseudo-molecular ion ([M + H]^+^ or [M + Na]^+^), Δ (mass error), RDB (relative double-bond equivalents).

**Table 4:** Acute and chronic toxicity values (fish/daphnid/green algae) for CLO and its identified TPs predicted in silico using ECOSAR v2.0.

Compound	Acute Toxicity (LC_50_/EC_50_)			Chronic Toxicity (ChV)		
(Chemical Category)	Fish LC_50_ (mg L^−1^)	Daphnid LC_50_ (mg L^−1^)	Green Algae EC_50_ (mg L^−1^)	Fish ChV (mg L^−1^)	Daphnid ChV (mg L^−1^)	Green Algae ChV (mg L^−1^)
CLO (AA)	372.6	37.94	42.76	35.07	2.67	12.67
CLO (NN)	354.2	105.7	63.50	177.0	5.91	3.48
TP1 (PY/DZ)	189.1	64.45	3.00	0.42	1.11	1.23
TP2 (AA)	300.9	30.67	34.48	28.22	2.16	10.23
TP2 (NI)	7.86	0.25	92.78	3.68	0.03	9.89
TP3 (AA)	1694	152.1	219.9	234.0	9.49	59.50
TP3 (NI)	18.67	0.27	489.6	18.27	0.07	33.53
TP4 (AA)	74.33	8.31	7.78	5.26	0.64	2.47
TP4 (NI)	3.64	0.21	24.03	1.00	0.02	3.53
TP5 (SU)	634.8	407.2	0.86	12.99	14.68	0.30
AA: aliphatic amines	Very toxic: LC_50_/EC_50_/ChV ≤ 1 mg L^−1^					
NN: neonicotinoids	Toxic: 1 mg L^−1^ < LC_50_/EC_50_/ChV ≤ 10 mg L^−1^					
SU: substituted ureas	Harmful: 10 mg L^−1^ < LC_50_/EC_50_/ChV ≤ 100 mg L^−1^					
NI: nicotinoids	Not harmful: 100 mg L^−1^ < LC_50_/EC_50_/ChV					
PY/DZ: pyrroles/diazoles						

The background color for each predicted value indicates the category to which that value corresponds (i.e., Very Toxic—Red, Toxic—Yellow, Harmful—Blue, Not Harmful—Green).

## Data Availability

Data are contained within the article.
